# Congenital Retroversion of the Uterus and Pregnancy

**Published:** 1907-06-29

**Authors:** 


### Gynæcology.

### CONGENITAL RETROVERSION OF THE UTERUS AND PREGNANCY.

### The Relations existing between them.

Cases of premature expulsion of the ovum in first pregnancies are sufficiently frequent to make it of some interest to inquire into their possible causation. Among these causes syphilis has always been looked upon as of paramount importance, but in actual practice it cannot be said to account for more than a very small percentage. In private practice it is usually easy to discover the syphilitic cases, because the husbands are accessible; in hospital practice this is rarely possible, so that some element of doubt as to the true cause of the abortion will remain. Apart from syphilis, however, the causes of abortion in first pregnancies are not always clear. The subjects are often apparently in robust health, and have not suffered from any obvious disease of the generative, circulatory, or nervous systems. The patients themselves, on the other hand, seldom have any doubt as to the cause of the abortion, and they will usually attribute it to some trivial accident or some mental disturbance. Viewed from the critical standpoint of the pathologist this hypothesis is often without foundation. Whilst not discrediting the possibility of a severe accident causing abortion, we cannot believe that the usual trivial accidents assigned as causes, can have such an effect in the absence of some pathological lesion of the uterus.

#### Endometritis and Uterine Congestion.

The commonest antecedent of abortion, there can be no doubt, is uterine congestion and endometritis, and, given such a predisposing factor, a very small disturbance of the pregnant uterus will be sufficient to cause a slight hæmorrhage into the chorio-decidual space. This hæmorrhage acts as a foreign body, stimulates the uterus to powerful contraction, and sooner or later the ovum is expelled. When, however, an apparently healthy woman aborts in her first pregnancy we must look, as a rule, for some other cause of the occurrence. We fully admit that some young women have already uterine congestion and endometritis before marriage, and that this may predispose to abortion as in multiparæ, but such cases are exceptional. There is, however, a group of cases, some examples of which have come before the writer, which it may be of interest to consider, and which do not fall into any of the ordinary categories of the causes of abortion. These cases are associated with congenital retroversion of the uterus. Although the congenital nature of these displacements is not strictly capable of proof, it is probable for several reasons. First, their possessors have not gone through a pregnancy, which we know is the commonest cause of acquired backward displacements; secondly, these uteri are often undersized, though of the adult type, and are associated with menstrual disturbances. Thirdly, there is frequently a very short posterior vaginal wall, which is an absolute bar to replacement, and retention in the normal position by pessaries.

Menorrhagia is quite uncommon in such cases, and only occurs when the uterus becomes pathologically congested. In spite, however, of these disadvantages, they appear to militate only slightly against conception, probably because the endometrium is normal and the walls in contact as they should be in a virgin uterus. When, however, pregnancy has advanced to about two months, for no apparent reason abortion occurs. It has nothing to do with incarceration in the pelvis, because the uterus is not large enough to become incarcerated. It has also nothing to do with congestion, because there have been no previous signs of congestion. After abortion has occurred involution of the uterus takes place normally as a rule, and that no congestion nor endometritis results is shown by the absence of menorrhagia. Even after abortion all attempts at replacement fail; the normal position, in fact, of such uteri is retroversion. The important point we wish to emphasise in the clinical aspect of these cases is this: that if pregnancy can be demonstrated within the first two months, it will be found to be quite easy to prevent abortion by replacing the uterus and keeping it in its place. Whilst in the unimpregnated condition it is impossible to maintain an anteverted position by means of pessaries, because of the tension so put upon the short posterior vaginal wall; in the pregnant condition this is not the case. The tissues of the vagina become relaxed and softened by pregnancy, and it is quite easy to replace, and, more important still, to keep in place the gravid uterus by a small ring pessary.

#### Some Conclusions.

Where abortion has occurred once already, and the displacement is discovered, the patient should be advised to come at once for treatment if pregnancy is again suspected. In cases so treated the results are good, and pregnancies go to full term. The retroversion recurs after labour, but gives rise to no symptoms, showing that involution occurs normally. It must be clearly understood that these remarks apply only to a small group of cases, namely, those in which pregnancy occurs in a congenitally retroverted uterus. Where retroversion of the uterus is acquired, congestion and endometritis nearly always accompany it, with their respective symptoms. Pregnancy in such uteri commonly gives rise to the well-known vesical symptoms of the retroverted gravid uterus, and a certain proportion of such cases abort again and again. In this case, however, the endometritis is really to blame, and not the displacement. If there happens to be no endometritis, there is not so great a predisposition to abortion as in the congenital cases.

